# Supplementary material for: Structural basis for hyperpolarization-dependent opening of human HCN1 channel
Source: Nat Commun. 2024 Jun 18;15:5216. doi: 10.1038/s41467-024-49599-x (PMC11189445; doi:10.1038/s41467-024-49599-x)
Supplement: Supplementary file 1 — Supplementary Information [file 41467_2024_49599_MOESM1_ESM.pdf]

SUPPLEMENTARY FIGURES AND TABLES

Structural Basis for Hyperpolarization-dependent Opening of Human HCN1 Channel.

Verena Burtscher[1,3*], Jonathan Mount[2,3,7*], Jian Huang[9], John Cowgill[1,3,6], Yongchang Chang[1,3], Kathleen Bickel[1,3], Jianhan Chen[9], Peng Yuan[2,3,7,8], Baron Chanda[1,3,4,5]

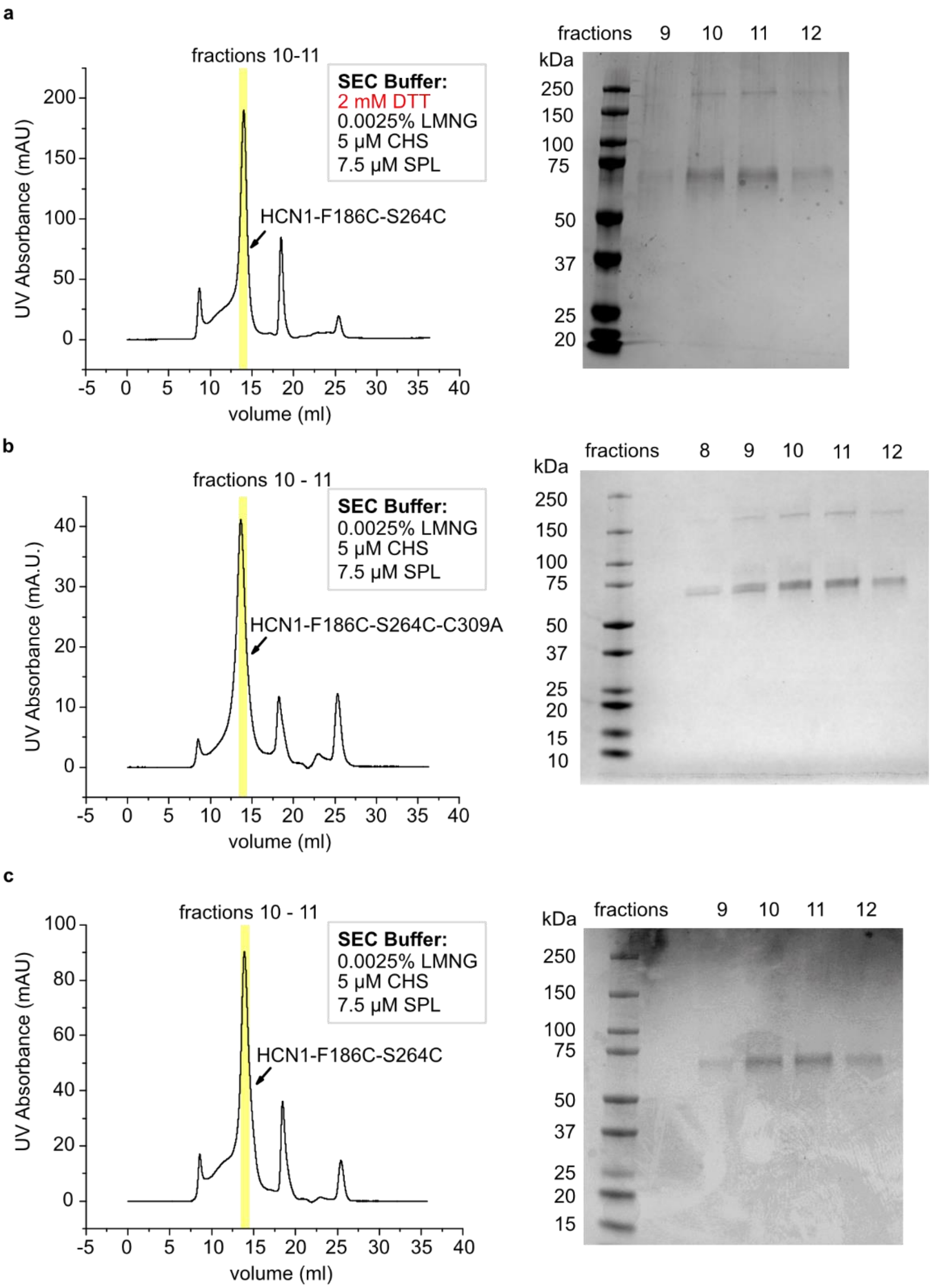

Supplementary Fig. 1: Representative size-exclusion chromatographs and Coomassie blue stained SDS-PAGE gels of the protein samples for obtaining the Closed, Intermediate and Open conformation. **a**, The Closed state was obtained from HCN1-EM F186C-S264C purified in LMNG/CHS/SPL in the presence of the reducing agent, dithiothreitol (DTT). **b**, The Intermediate state was obtained from HCN1-EM F186C-S264C-C309A purified in LMNG/SPL without DTT. **c**, The Open state was obtained from HCN1-

EM F186C-S264C purified in LMNG/CHS/SPL without DTT. In all cases, fractions 10 & 11 (yellow) were pooled and used for grid freezing.

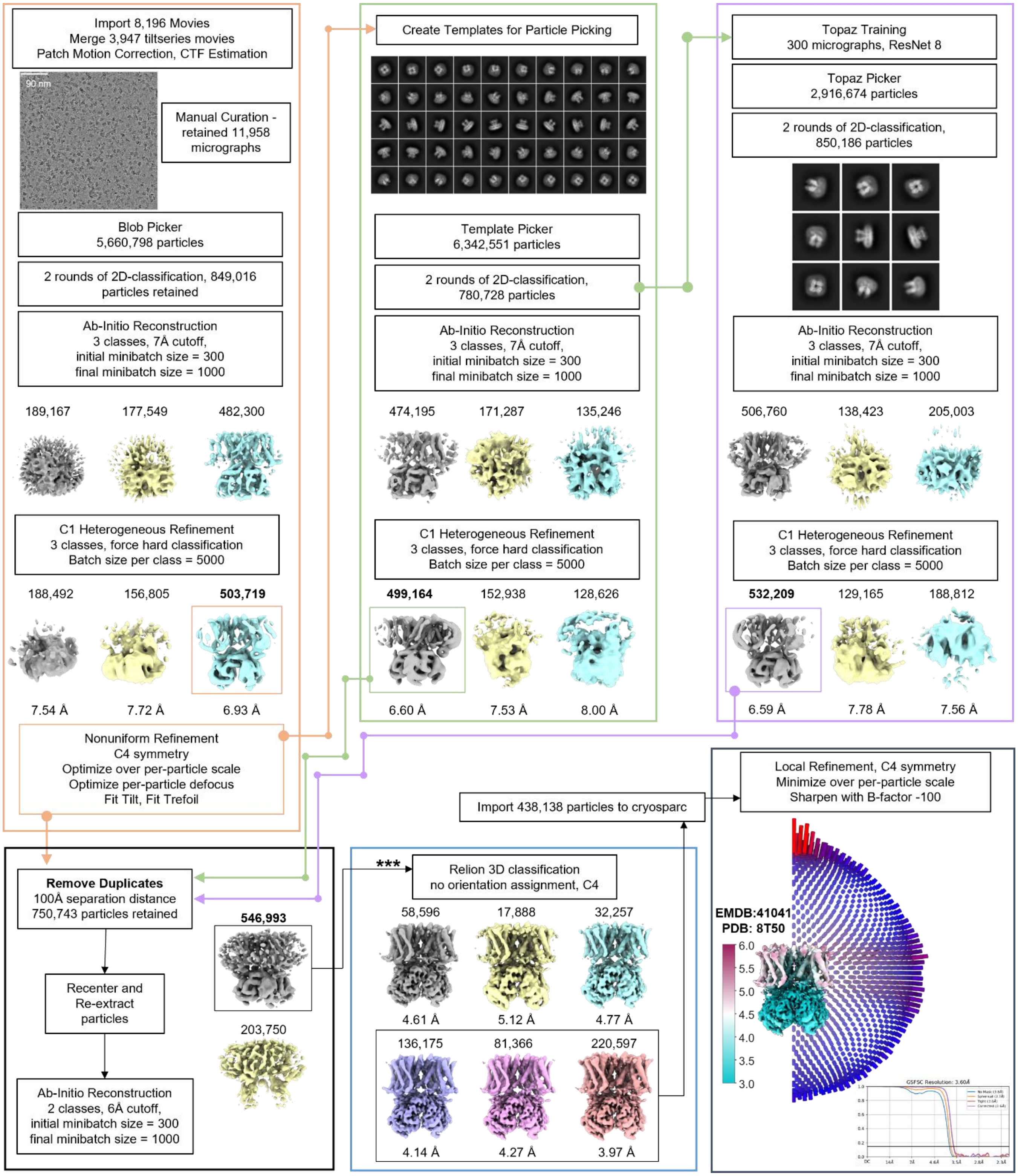

Supplementary Fig. 2: Cryo-EM processing workflow for the cAMP bound HCN1 Open conformation (HCN1-EM F186C-264C).

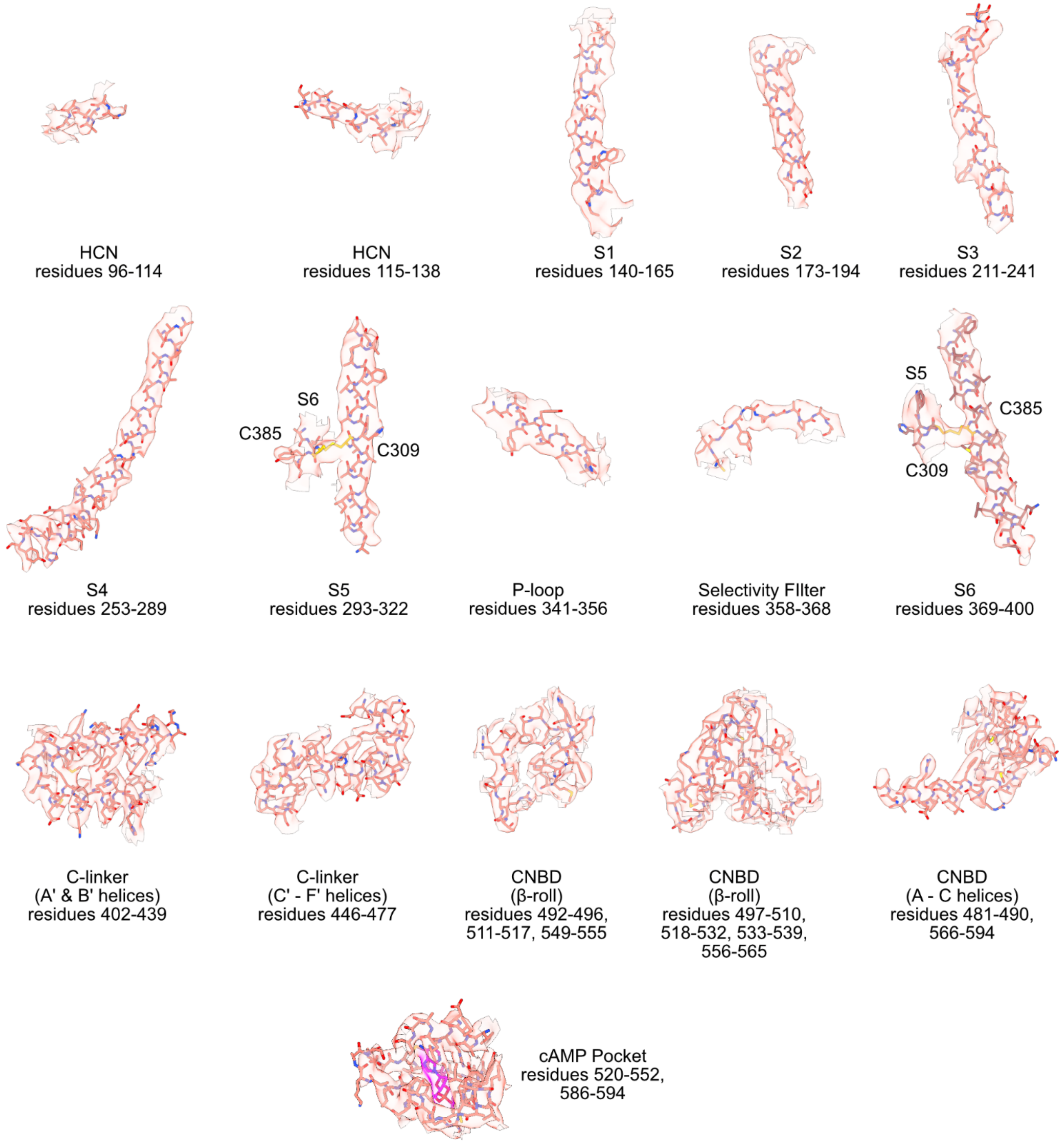

Supplementary Fig. 3: Cryo-EM density of cAMP-bound Open-state structure of HCN1-EM F186C-S264C. Structure and cryo-EM density maps are depicted for the regions indicated.

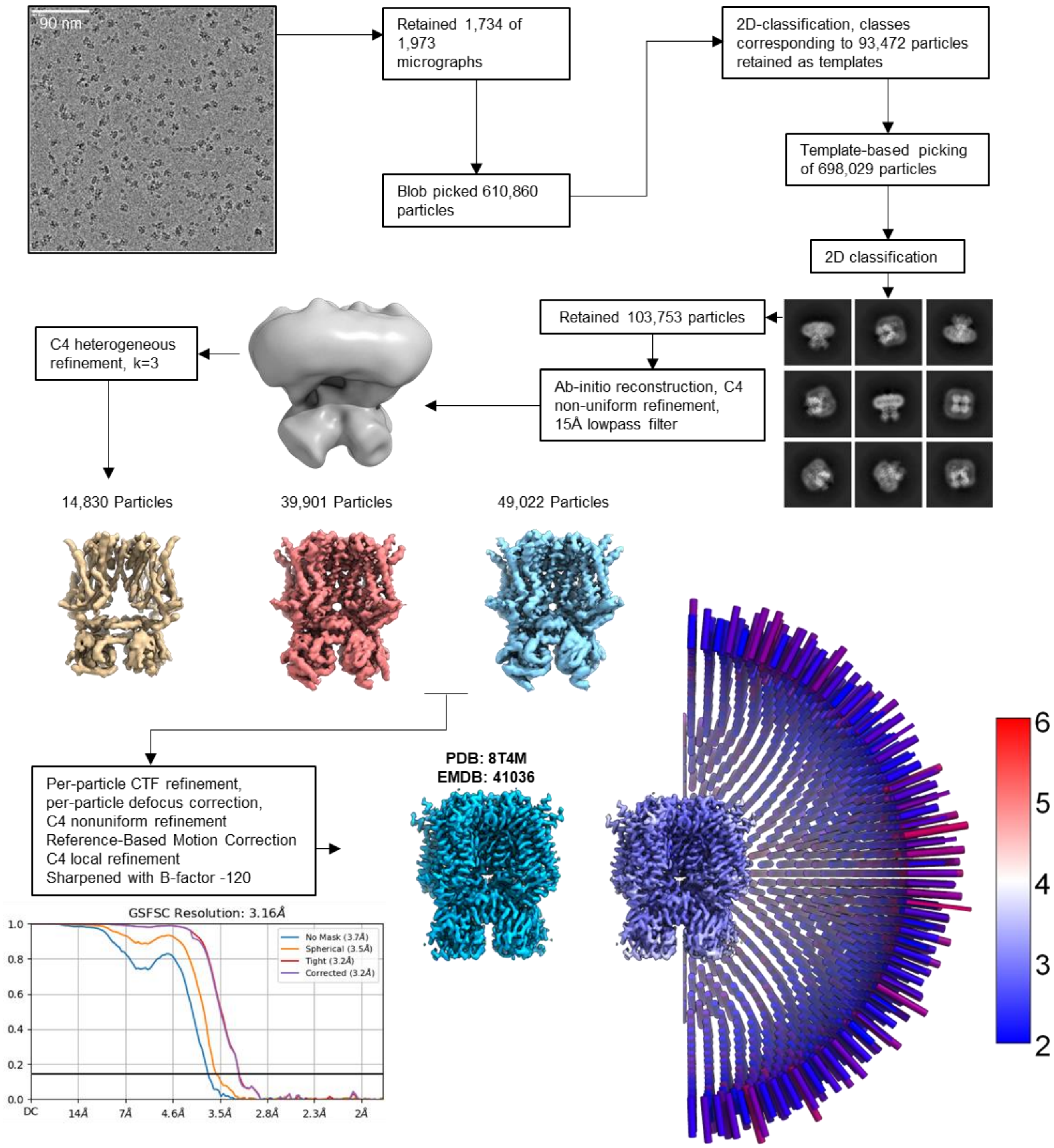

Supplementary Fig. 4: Cryo-EM processing workflow for the cAMP bound HCN1 Closed conformation (HCN1-EM F186C-264C).

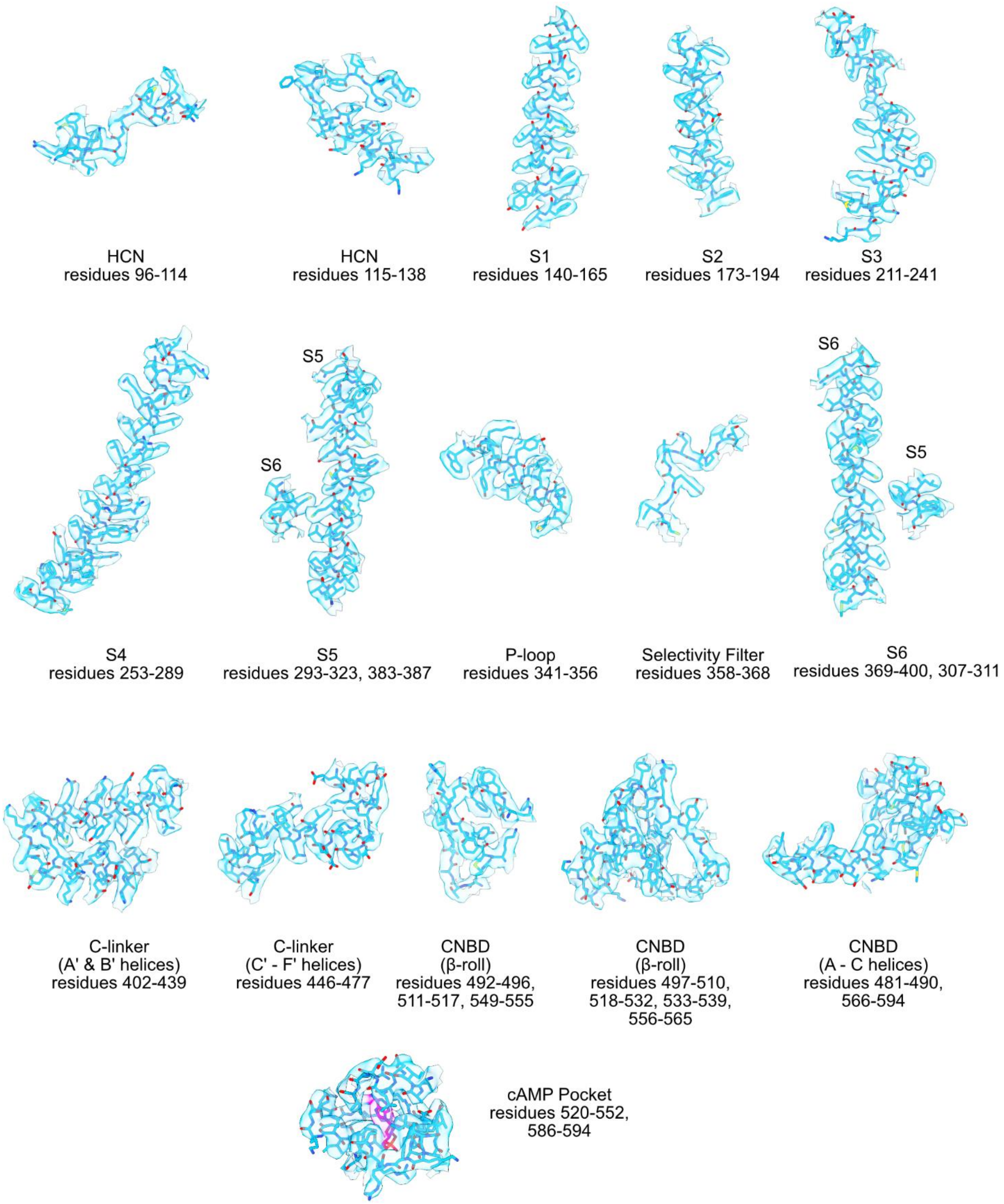

Supplementary Fig. 5: Cryo-EM density of cAMP-bound Closed-state structure of HCN1-EM F186C-S264C. Structure and cryo-EM density maps are depicted for the regions indicated.

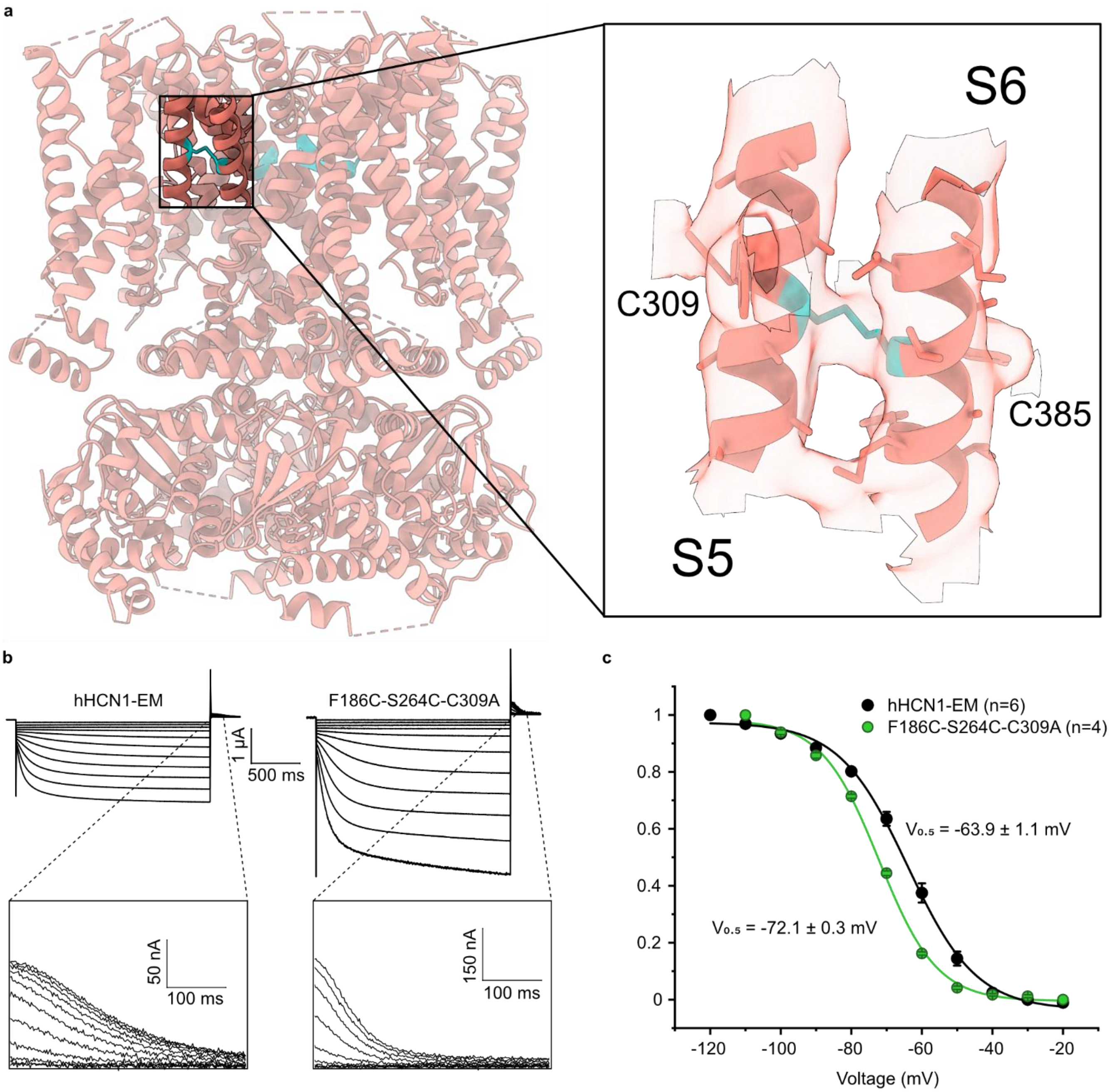

Supplementary Fig. 6: Disulfide bridge marginally stabilizes the Open state structure. **a,** Ribbon presentation of the Open state structure showing the location of the disulfide bond between C309 and C385. Inset: Close-up of the disulfide bond and corresponding electron density. **b,** *h*HCN1-EM-F186C-S264C-C309A channels activate at more negative potentials and have faster deactivation kinetics than *h*HCN1-EM. Representative sample traces of HCN1-EM (left panel) and F186C-S264C-C309A (right panel) and corresponding close-ups of the tail currents. **c**, Conductance-voltage curves of F186C-S264C-C309A channels compared to HCN1-EM. Data are given as mean ± SE.

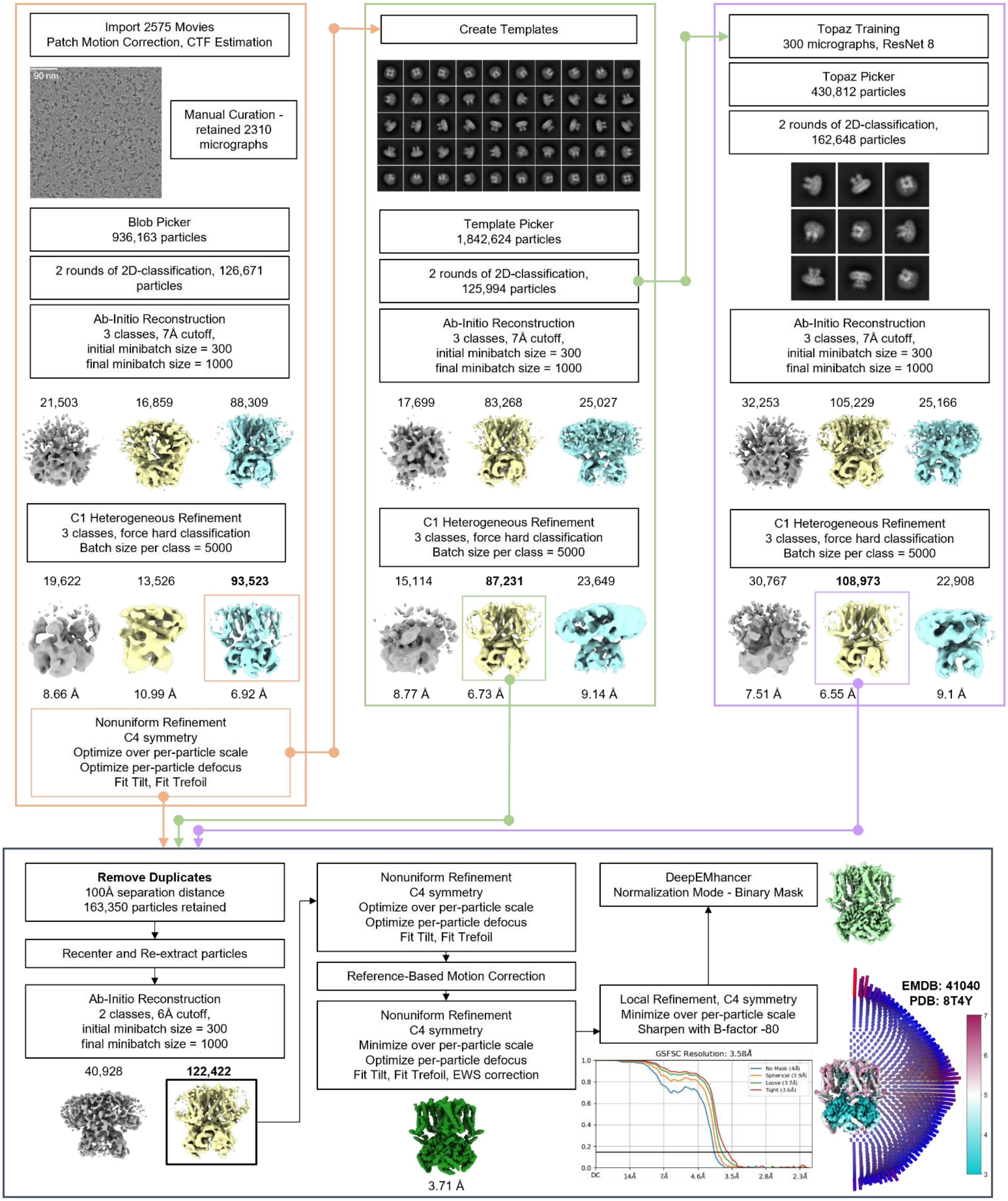

Supplementary Fig. 7: Cryo-EM processing workflow for the cAMP bound HCN1 Intermediate conformation (HCN1-EM F186C-S246C-C309A).

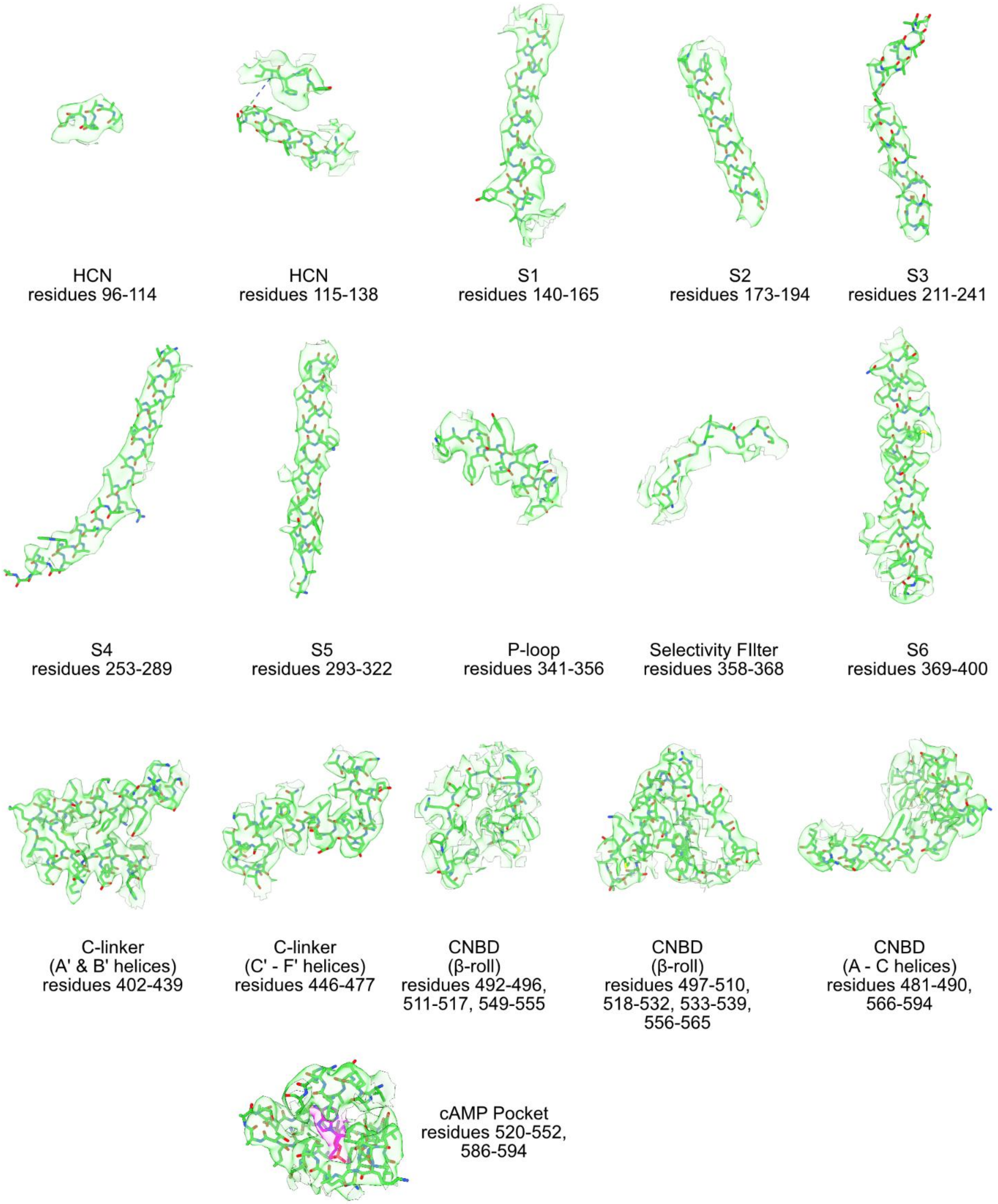

Supplementary Fig. 8: Cryo-EM density of cAMP-bound Intermediate-state structure of HCN1-EM F186C-S264C-C309A. Structure and cryo-EM density maps are depicted for the regions indicated.

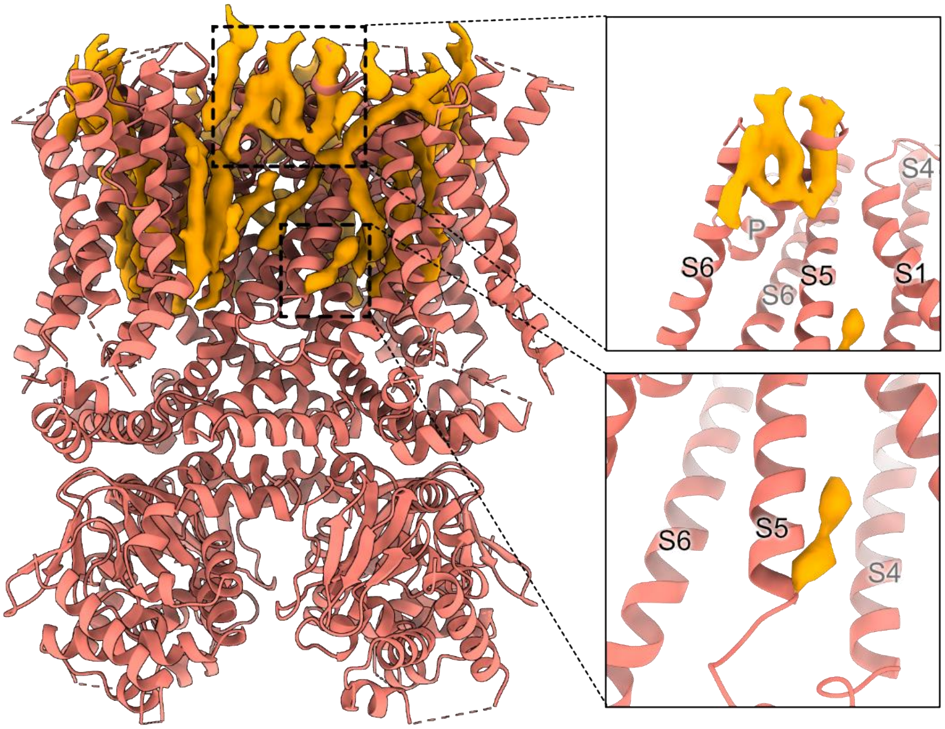

Supplementary Fig. 9: Annular lipids in the Closed state structure (orange) superimposed onto the Open structure (salmon) indicate overlap with the P- helix (upper inset) and the S5 helix (lower inset). The lipids observed in the Closed structure would sterically clash with the Open channel conformation, indicating that there would be displacement of bound lipids during channel gating.

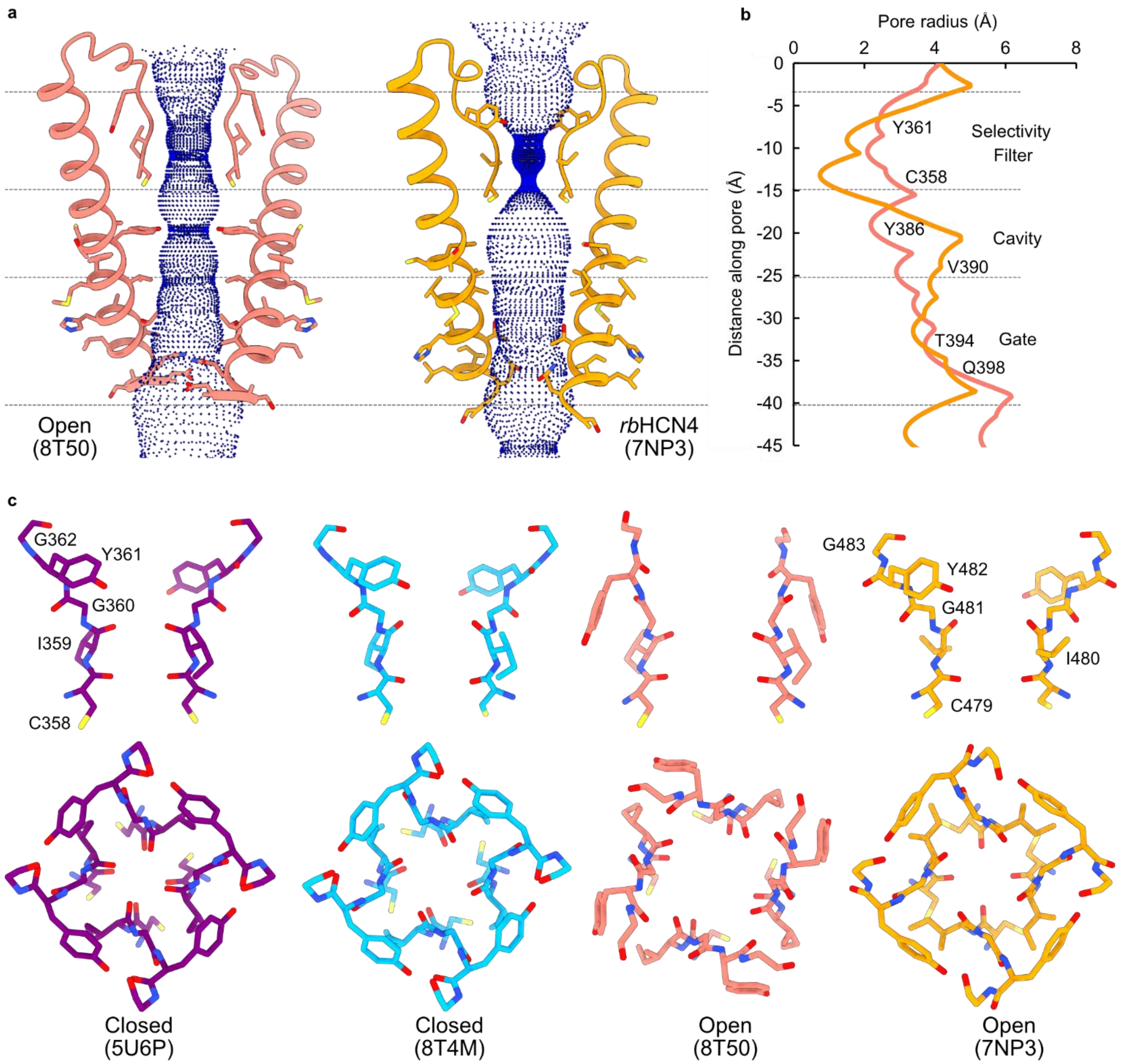

Supplementary Fig. 10: Ion conduction pathways of open HCN1 and HCN4. **a** Comparison of the solvent-accessible pathways of the open states of human HCN1 (8T50) and rabbit HCN4 (7NP3). Left panel, the pore lining S6 helices, and the selectivity filter of two opposing subunits (ribbon) are depicted together with the corresponding solvent-accessible pathway (blue dots) generated by the HOLE program. Residues comprising the pore are shown as sticks. Right panel, plot of the pore radii for both structures. **b,** Comparison of the selectivity filters between the pre-existing Closed state structure of HCN1 (5U6P) and the Open state structure of HCN4 (7NP3), with our Closed and Open state structures.

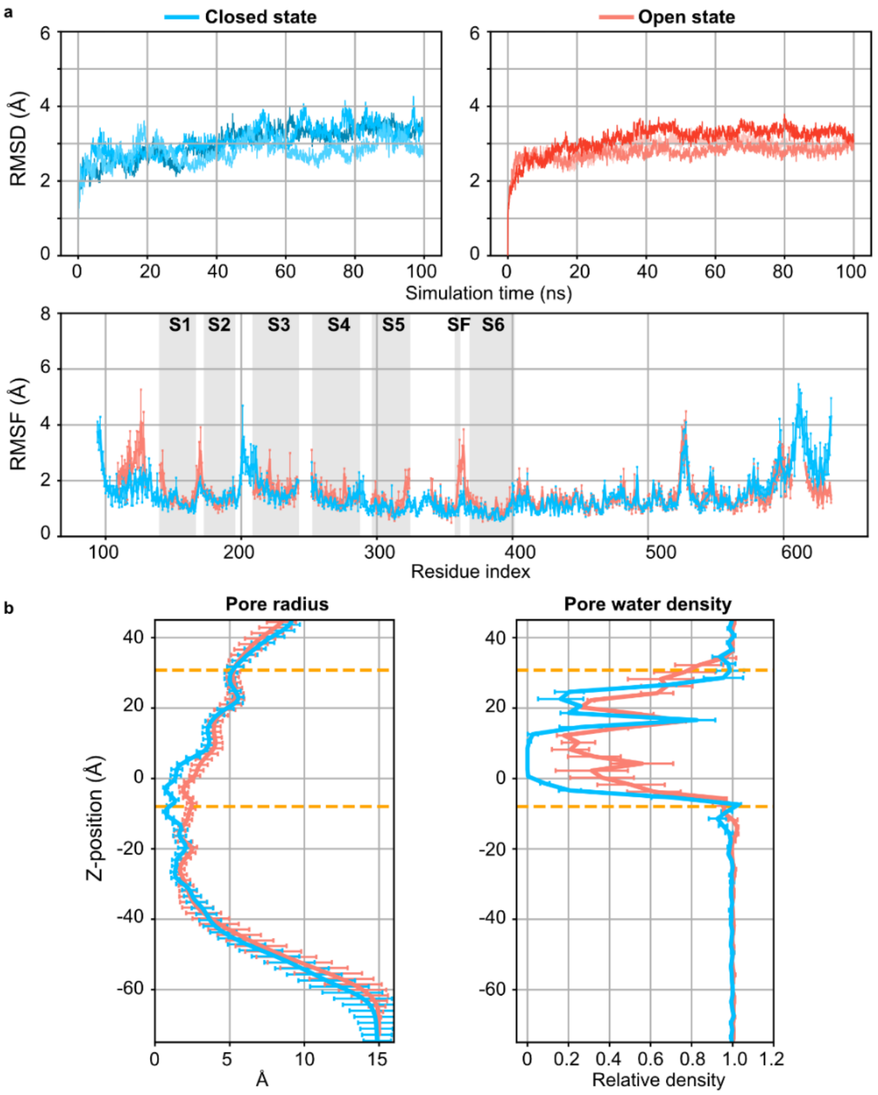

Supplementary Fig. 11: Stability and pore properties of the open and close states of the HCN1 channel. **a,** The protein backbone RMSD during three parallel 100 ns unrestrained simulations and the averaged residue RMSF profiles of the open (red lines) and closed (blue lines) states. The locations of TM helices (S1-S6) and the selectivity filter region (SF) are marked in the lower panel. The RMSF was averaged over four monomers in the simulation and the error bars show the standard deviations for all four monomers in the simulations. **b,** Averaged pore radius (left) and pore water density profile (right) derived from the unrestrained simulations of the open (the salmon line) and closed (the blue line) states. The center of mass of the bundle-crossing lower gate (residue 394-398) is set to be z = 0 Å. The error bars are the standard deviations calculated from the three parallel unrestrained simulations. The locations of the membrane interfaces are indicated by the yellow dashed lines.

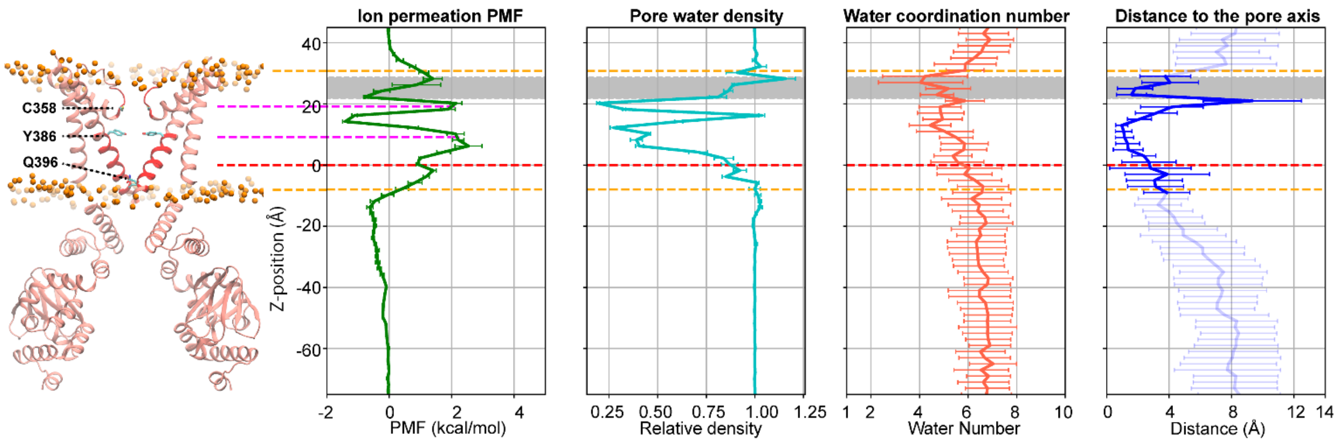

Supplementary Fig.12. Estimated ion permeation free energy profile (potential of mean force, PMF), the hydration level along the z-position, the water coordination number, and the offset to the pore axis during permeation calculated from equilibrium simulations. The bundle-crossing lower gate (residue 394-398) of the HCN1 channel is set to be z = 0 Å. The representations of the diagonal monomers are shown in cartoon mode, and the lipid phosphate atoms of the POPC membrane are shown as orange spheres (in the left panel), shown in scale with the middle and right panels. Error bars in the free energy and pore water density profiles are the standard deviations calculated from three parallel simulations. A cutoff of 3.5 Å was used to calculate the first shell hydration (water coordination number). Distance to the pore axis during permeation shows the deviations of potassium ions with respect to the pore central axis, and it is only meaningful in the transmembrane region indicated by the two orange dashed lines. Positions of the membrane interfaces, bundle-crossing gate, and key barriers in the PMF profile are marked using dashed lines colored in orange, red, and magenta, respectively. The gray stripes mark the SF region. In addition, the three pore-lining residues (C358, Y386 and Q396) corresponding to the energy barriers are also shown using sticks in the left panel.

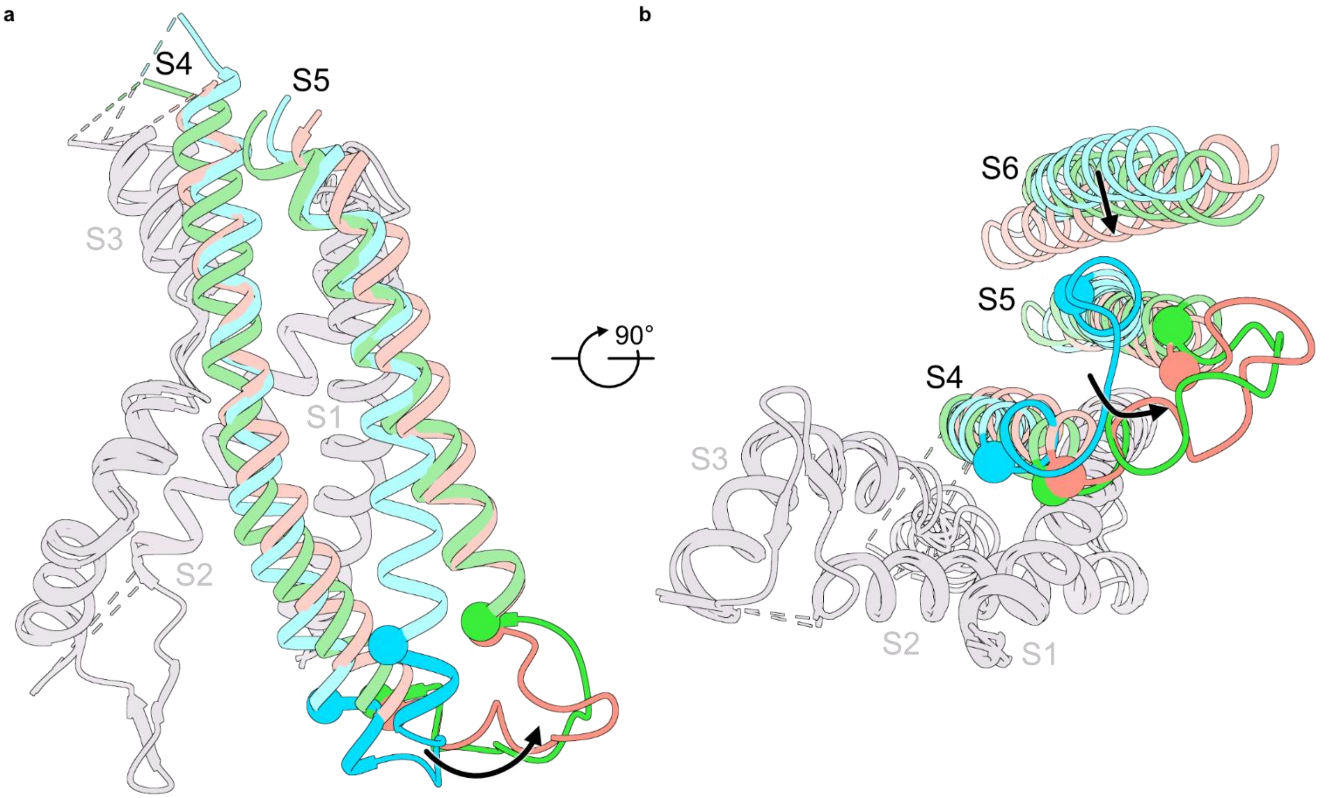

Supplementary Fig. 13: Conformational changes in the EM coupling interface of Closed, Intermediate and Open state. **a,** Sideview of transmembrane helices S1-S5. Color coding is the same as in Fig.1. I284 and V296 are shown as spheres, and the highlighted region between these two residues undergo a helix-coil transition. The P-loop and the S6 transmembrane helices were not depicted for clarity. **b,** Bottom view of transmembrane helices S1-S6 of Closed, Intermediate and Open states. The structures were aligned to S1 and S2 helices.

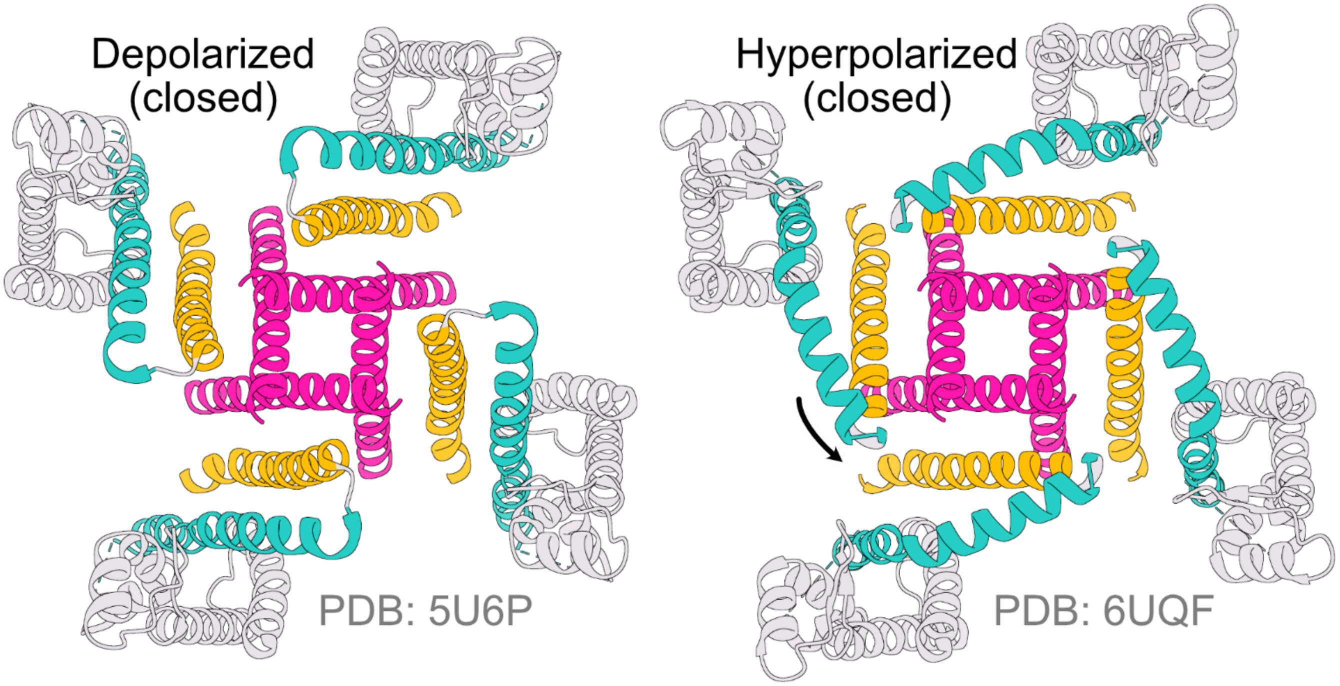

Supplementary Fig. 14: Relative motions of the EM coupling interface and pore helices in the pre-existing HCN1 structures. The bottom view of the transmembrane helices highlights the S6 helices in pink, S5 helices in yellow, and the S4 helices in turquoise. For clarity, the P-helices and selectivity filter have been omitted. The left panel shows the closed state (5U6P), whereas the right panel shows the pre-open state (6UQF). The arrow indicates the direction of motion of the intracellular end of the S4 segment.

Supplementary Table 1 | Cryo-EM data collection, refinement and validation statistics

	*h*HCN1 Closed<br>EMDB-41036<br>PDB 8t4m	*h*HCN1 Intermediate<br>EMDB-41040<br>PDB 8t4y	*h*HCN1 Open<br>EMDB-41041<br>PDB 8t50
**Data collection and processing**			
Magnification	150,000x	59,000x	59,000x
Voltage (kV)	200	300	300
Electron exposure (e–/Å^2)	53.08	53.3	54.55, 53.99, 51.24
Defocus range (μm)	-0.8 to -2.4	-0.8 to -2.4 μm	-0.8 to -2.4 μm
Pixel size (Å)	0.928	1.081	1.081
Symmetry imposed	C4	C4	C4
Initial particle images (no.)	698,029	936,163	2,916,674
Final particle images (no.)	88,923	122,422	438,138
Map resolution (Å)	3.16	3.58	3.60
FSC threshold	0.143	0.143	0.143
Map resolution range (Å)	2-4	3-6	3-6
**Refinement**			
Initial model used (PDB code)	5u6p	8t4m	6uqf
Model resolution (Å)	3.28	3.93	3.89
FSC threshold	0.5	0.5	0.5
Model resolution range (Å)	2.93-3.4	3.69-3.96	3.57-3.89
Map sharpening *B* factor (Å^2)	-120	-80	-100
Model composition			
Non-hydrogen atoms	17000	13348	12720
Protein residues	2132	2004	1896
Ligands	4	4	4
B factors (Å^2)			
Protein	84.65	167.21	118.77
Ligand	117.41	107.68	84.91
R.m.s. deviations			
Bond lengths (Å)	0.004	0.004	0.004
Bond angles (°)	0.927	1.029	0.979
Validation			
MolProbity score	1.53	1.69	1.64
Clashscore	5.78	7.50	7.66
Poor rotamers (%)	0.00	0.00	0.00
Ramachandran plot			
Favored (%)	96.6	95.94	96.54
Allowed (%)	3.40	4.06	3.46
Disallowed (%)	0.00	0.00	0.00

Supplementary Table 2: Conformation and bending angle of S4 helix in homologous CNG channels. Angles are calculated using anglebetweenhelices.py utilizing the Cα-fit preset.

Channel	Conductive State	S4 conformation	Bending Angle	PDB
*h*HCN1	Closed	Up	16.9°	8t4m
	Closed	Intermediate	29.4°	8t4y
	Closed	Down	60.7°	6uqf
	Open	Down	28.9°	8t50
*r*HCN4	Open	Up	14.4°	7np3
*h*EAG	Closed	Down	29.9°	8ep1
*c*TAX-4	Closed	Down	53.3°	6wej
*c*TAX-4^{R421W}	Open	Down	54.9°	7n17
