# Supplementary material for: Structural basis for hyperpolarization-dependent opening of human HCN1 channel
Source: Nat Commun. 2024 Jun 18;15:5216. doi: 10.1038/s41467-024-49599-x (PMC11189445; doi:10.1038/s41467-024-49599-x)
Supplement: Supplementary file 3 — Description of Additional Supplementary Files [file 41467_2024_49599_MOESM3_ESM.pdf]

**Description of Additional Supplementary Files**

**File Name:** Supplementary Movie 1

**Description:** All-atoms MD-simulation of potassium permeation through the Open state pore. Region comprising residues 297- 400, which include the S5-helix, Phelix, selectivity filter, and the S6 helix from two diagonal protomers, are depicted in the cartoon representation. Spheres represent potassium ions. To demonstrate the permeation event, we highlighted one potassium ion in green. The red region in the cartoon denotes the restrained areas during the simulation. Critical pore-lining residues in the filter, along with Y386 and water molecules within a specified cutoff radius of 12 Å around the central pore axis, are all depicted using the stick representation.

**File Name:** Supplementary Movie 2

**Description:** Interface between the S4/S5-linker and C-linker of the Closed state. Cryo-EM map and structure are depicted. The region that undergoes a helix-to-coil transition in the Open state is highlighted in yellow. For comparison, see Supplementary Movie 2.

**File Name:** Supplementary Movie 3

**Description:** Interface between the S4/S5-linker and C-linker of the Open state. Cryo-EM map and structure are depicted. The region that undergoes a helix-to-coil transition is highlighted in yellow. For comparison with the Closed state, see Supplementary Movie 1.

**File Name:** Supplementary Movie 4

**Description:** Conformational rearrangement from Closed to Open state. The S4 helices, the voltage sensors, are shown in turquoise, the S5 helices are shown in gold and the pore-lining S6 helices are highlighted in pink
