# Supplementary material for: Structural basis for hyperpolarization-dependent opening of human HCN1 channel
Source: Nat Commun. 2024 Jun 18;15:5216. doi: 10.1038/s41467-024-49599-x (PMC11189445; doi:10.1038/s41467-024-49599-x)
Supplement: Supplementary file 9 — Source Data [file 41467_2024_49599_MOESM9_ESM.zip › Source data/Chanda_MD files.docx]

Initial configuration of the Closed state in allatoms MD simulation.

Initial configuration of the Open state in allatoms MD simulation.

Final configuration after 100 ns of simulation of the Closed state in all-atoms MD simulation in the absence of an electric field.

Final configuration after 500 ns simulation of the Open state in all-atoms MDsimulation at an applied electric field of -500 mV.
