# Supplementary material for: Structural basis for hyperpolarization-dependent opening of human HCN1 channel
Source: Nat Commun. 2024 Jun 18;15:5216. doi: 10.1038/s41467-024-49599-x (PMC11189445; doi:10.1038/s41467-024-49599-x)
Supplement: Supplementary file 9 — Source Data [file 41467_2024_49599_MOESM9_ESM.zip › Source data/Chanda_Source data items.docx]

Source data items

Supplementary Data 1: Uncropped micrograph of a representative SDSPAGE gel of the fractions from the purified Closed state, as shown in Supplementary Figure 1a. Supplementary Data 2: Uncropped micrograph of a representative SDSPAGE gel of the fractions from the purified Intermediate state, as shown in Supplementary Figure 1b. Supplementary Data 3: Uncropped micrograph of a representative SDSPAGE gel of the fractions from the purified Open state, as shown in Supplementary Figure 1c. Supplementary Data 4: Source Data of representative sample traces and activation curves of hHCN1-EM-pUNIV or hHCN1-EM-F186C-S264C-C309A, as displayed in Supplementary Figure 6b-c
