# Supplementary figures and images for: Structural basis for hyperpolarization-dependent opening of human HCN1 channel
Source: Nat Commun. 2024 Jun 18;15:5216. doi: 10.1038/s41467-024-49599-x (PMC11189445; doi:10.1038/s41467-024-49599-x)

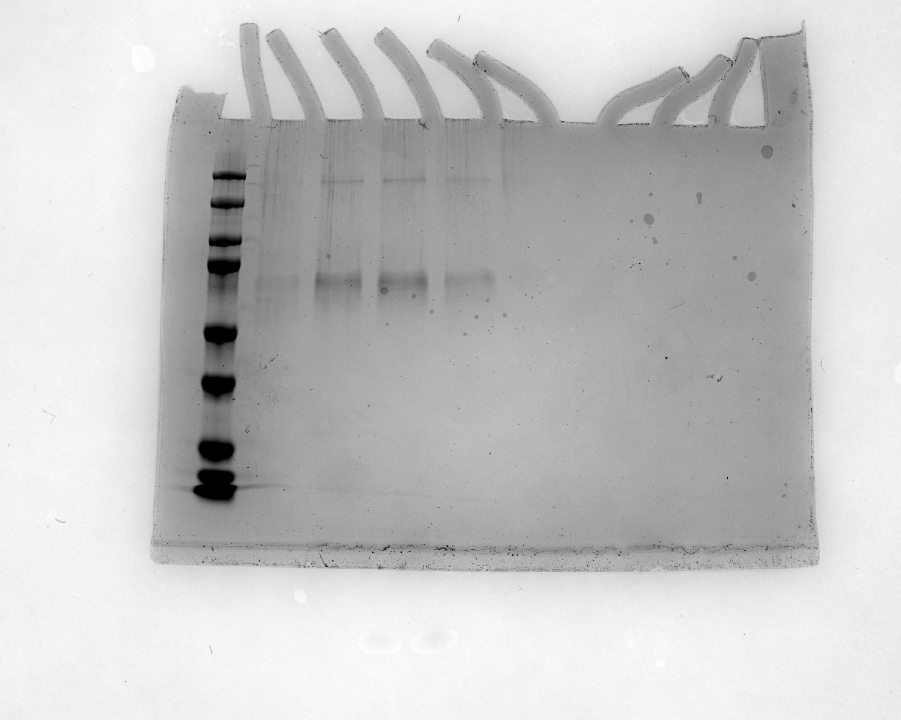

Supplement: Supplementary file 9 — Source Data [file 41467_2024_49599_MOESM9_ESM.zip › Source data/Source data 1.tif]

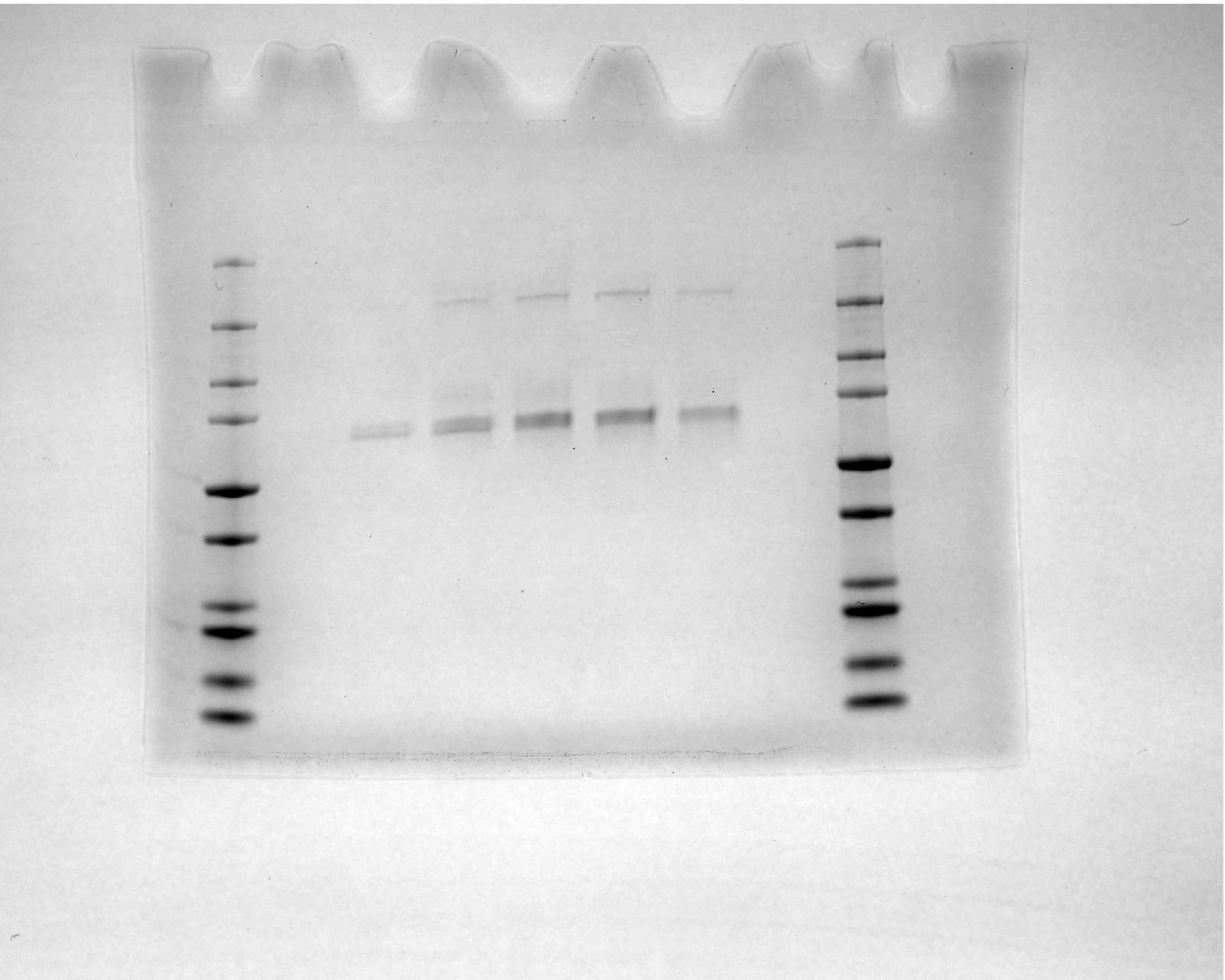

Supplement: Supplementary file 9 — Source Data [file 41467_2024_49599_MOESM9_ESM.zip › Source data/Source Data 2.tif]

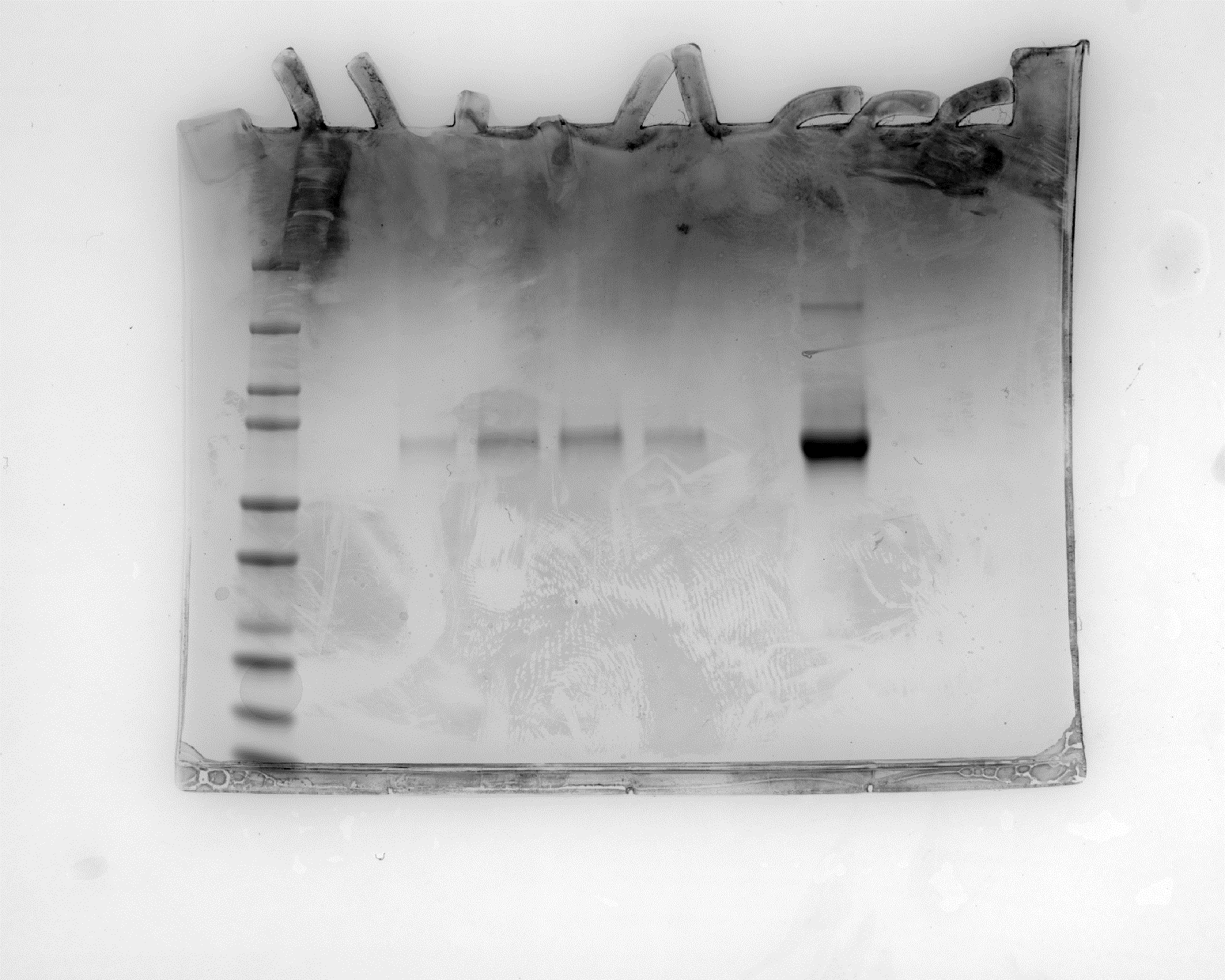

Supplement: Supplementary file 9 — Source Data [file 41467_2024_49599_MOESM9_ESM.zip › Source data/Source Data 3.tif]
